# Celastrol and Resveratrol Modulate *SIRT* Genes Expression and Exert Anticancer Activity in Colon Cancer Cells and Cancer Stem-like Cells

**DOI:** 10.3390/cancers14061372

**Published:** 2022-03-08

**Authors:** Helena Moreira, Anna Szyjka, Justyna Grzesik, Katarzyna Pelc, Magdalena Żuk, Anna Kulma, Fathi Emhemmed, Christian D. Muller, Kazimierz Gąsiorowski, Ewa Barg

**Affiliations:** 1 Department of Basic Medical Sciences, Faculty of Pharmacy, Wroclaw Medical University, 50-367 Wroclaw, Poland; anna.szyjka@umw.edu.pl (A.S.); justyna.grzesik91@gmail.com (J.G.); ewa.barg@umw.edu.pl (E.B.); 2 Department of Genetic Biochemistry, Faculty of Biotechnology, University of Wroclaw, 50-137 Wroclaw, Poland; pelc.katarzyna@yahoo.com (K.P.); magdalena.zuk@uwr.edu.pl (M.Ż.); anna.kulma@uwr.edu.pl (A.K.); 3 IPHC, UMR 7178 CNRS, Faculty of Pharmacy, University of Strasbourg, 67081 Illkirch, France; fathi.emhemmed@iphc.cnrs.fr (F.E.); cdmuller@me.com (C.D.M.)

**Keywords:** cancer stem cells, colon cancer, celastrol, resveratrol, sirtuins

## Abstract

**Simple Summary:**

The recovery rate in patients with metastatic colorectal cancer (CRC) remains low and declines with successive lines of treatment. This phenomenon is caused by the development of drug resistance and the presence of colorectal cancer stem cells (CSCs). Phytochemicals, like -celastrol and resveratrol, are very promising for colon cancer therapy, owing to their low or no toxicity and their pleiotropic activity, enabling them to interact with various biological targets. In the present study, the potential anticancer mechanisms of both compounds against metastatic colon cancer cells and the capacity to eradicate CSCs were investigated.

**Abstract:**

Metastatic colorectal cancer (CRC) remains a hard-to-cure neoplasm worldwide. Its curability declines with successive lines of treatment due to the development of various cancer resistance mechanisms and the presence of colorectal cancer stem cells (CSCs). Celastrol and resveratrol are very promising phytochemicals for colon cancer therapy, owing to their pleiotropic activity that enables them to interact with various biological targets. In the present study, the anticancer activities of both compounds were investigated in metastatic colon cancer cells (LoVo cells) and cancer stem-like cells (LoVo/DX). We showed that celastrol is a very potent anti-tumor compound against metastatic colon cancer, capable of attenuating CSC-like cells at the molecular and cellular levels. In contrast, resveratrol has a much greater effect on colon cancer cells that are expressing standard sensitivity to anticancer drugs, than on CSC-like cells. In addition, both polyphenols have different influences on the expression of *SIRT* genes, which seems to be at least partly related to their anti-tumor activity.

## 1. Introduction

Despite the increasing number of treatment options available over the last years, colorectal cancer (CRC) is still one of the most therapy-resistant solid tumors. Treatment failure and disease relapse in patients with CRC are associated with the presence of intrinsic and/or acquired resistance of cancer cells. In addition to the well-described concept, referred to as multidrug resistance (MDR), resistance can be conferred by changes within the cell itself, by the acquisition of malignant molecular and cellular modifications that alter drug sensitivity [1,2]. More recently, resistance to therapy and cancer recurrence have been attributed to the presence of cancer stem cells (CSCs) within the tumor mass [3]. The CSCs population is associated with either the accumulation of genetic and epigenetic alterations in colorectal stem cells and normal tumor cells, or the dedifferentiation of somatic cells caused by various genetic and environmental factors. Thus, the CSCs seem to be a phenotypically and functionally heterogeneous dynamic population within the tumor [1]. CSCs are less responsive or fully unresponsive to conventional therapies that target primarily proliferating neoplastic cells, while CSCs remain dormant. In the present state of knowledge, it is undeniable that tailored therapeutic protocols should aim at both killing proliferating cells and eliminating CSCs [4]. Therefore, designing a new therapeutic strategy to aid the elimination of CSCs is imperative to improve the treatment of colorectal cancer.

**Figure 1:**
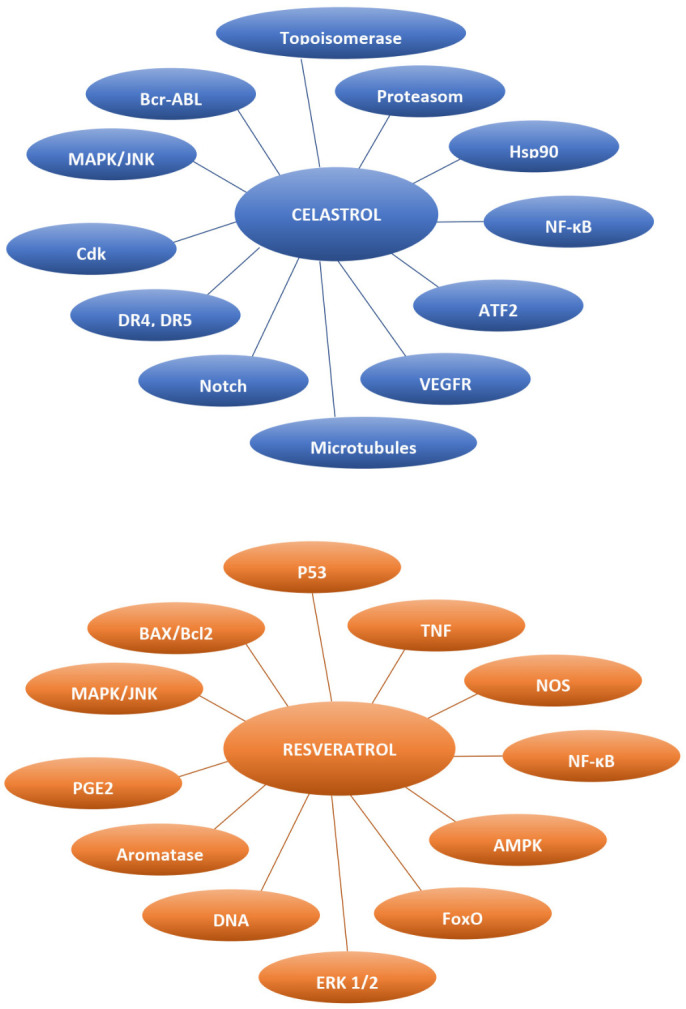
Molecular targets modulated by celastrol and resveratrol (Adapted from [10,11]).

In the last decades, naturally occurring compounds have gained considerable attention as potential cancer therapeutics with low potential for inducing side effects. In addition, several recent reports show that phytochemicals, due to the ability to target multiple signaling pathways, may affect the viability of CSCs [5]. Natural products constitute a wide range of organic compounds produced by the pathways of secondary metabolism. The general structural classes of secondary metabolites include alkaloids, tannins, flavonoids, and terpenoids [6]. Celastrol is a pentacyclic triterpenoid that belongs to a category of triterpene quinine methides. It can react with nucleophilic thiol groups of cysteine residues in many proteins that influence their function. Celastrol (tripterine) was isolated from the root of *Tripterygium wilfordii,* a plant that originated from traditional Chinese medicine, named as Thunder of God Vine. This medicinal plant was generally used for the treatment of inflammatory and auto-immune diseases. However, it has a broad range of antitumor properties [7]. Resveratrol is a trihydroxy-trans-stilbene and belongs to the non-flavonoid polyphenol family of natural compounds. It is a phytoalexin, firstly isolated from the roots of the white hellebore (*Veratrum grandiflorum*). The dominant source of resveratrol is the root of *Polygonum cuspidatum*, a medicinal herb from Eastern-Asian countries like Japan, China, and Korea. It also appears in fruits and preserves, especially in grapes and red wine. This compound is described for its cardioprotective activity in the prevention of cardiovascular diseases. However, resveratrol has been demonstrated to be an effective chemotherapeutic and chemopreventive agent [8,9]. Both celastrol and resveratrol are functionally pleiotropic agents, acting on multiple targets including those involved in the cell cycle, proliferation, and apoptosis (Figure 1) [10,11], thus could potentially suppress CSCs’ survival. Several studies has connected these phytochemicals to the elimination of CSCs in some cancers [12,13,14,15,16].

Genomic instability and epigenetic alterations are key features of most cancers and CSCs. Sirtuins (SIRTs) are a class of epigenetic regulators, belonging to the histone deacetylases (HDACs) and are involved in major biological functions such as transcription, metastasis, autophagy, cell cycle, DNA damage repair, angiogenesis, stress responses, and senescence. SIRTs comprise seven members (SIRT1–7) with different cellular localization: nucleus (SIRT1,6,7); cytosol (SIRT2); and mitochondria (SIRT3,4,5). SIRTs have been shown to be dysregulated in tumor cells. Moreover, some evidence indicate that sirtuins, especially SIRT1 and SIRT2, might play an essential role in the maintenance and differentiation of CSCs. On the other hand, sirtuins are assigned a dual function as tumor suppressors and promoters, depending on their tissue- and cancer-specific expression, as well as experimental conditions [17,18,19].

Here we investigated the influence of celastrol and resveratrol on *sirtuins*’ gene expression in metastatic colon cancer cell lines: LoVo cells (with standard sensitivity to anticancer drugs) and LoVo/DX cells (with high resistance to doxorubicin). We showed that LoVo/DX cells present several important characteristics of CSCs. Furthermore, the anticancer mechanisms of both compounds were explored, including the effect on apoptosis, cell cycle, ROS status, DNA double-strand breaks, and DNA repair genes. We discuss the role of sirtuins in observed cell responses to these treatments as well. The tested sirtuins were selected on the basis of their role in cancer cells, i.e., SIRT 1 and 2 are associated with cancer stem cells; SIRT3 is an important mitochondrial deacetylase responsible for reducing oxidative stress and ROS production; SIRT6 has been proposed as a prognostic indicator and a potential therapeutic target in colon cancer.

## 2. Materials and Methods

### 2.1. Materials

DMEM/F12 (Dulbecco’s Modified Eagle’s Medium: Nutrient Mixture F-12), HBSS (Hank’s Balanced Salt Solution), FBS (fetal bovine serum), ultra-glutamine 1, and gentamicin sulfate were purchased from Lonza (Basel, Switzerland). TrypLE™ Express was obtained from Gibco (Waltham, MA, USA). Resveratrol (99% of purity), rhodamine 123 (2-(6-Amino-3-imino-3H-xanthen-9-yl)benzoic acid methyl ester), Hoechst 33342 (2′-(4-Ethoxyphenyl)-5-(4-methyl-1-piperazinyl)-2,5′-bi-1H-benzimidazole trihydrochloride), DCF-DA (2,7-dichlorofluorescin diacetate), NAC (N-Acetyl L-cysteine), DMSO (Dimethyl Sulfoxide), paraformaldehyde (PFA), propidium iodide, and DAPI (4′,6-Diamidino-2-phenylindole dihydrochloride) were purchased from Sigma Aldrich (St. Louis, MO, USA). Celastrol (98% of purity) was purchased from the Cayman Chemical Company (Ann Arbor, MI, USA). Ethanol 96% was obtained from Chempur (Poland). FcR blocking reagent was obtained from Miltenyi Biotec. FITC mouse anti-human CD44 (Clone G44-26), Accutase™ Cell Detachment, and FITC Annexin V Apoptosis Detection Kit were obtained from BD Biosciences (Franklin Lakes, NJ, USA). Phospho-histone H2A.X (Ser139) mouse monoclonal antibody (CR55T33) Alexa Fluor^®^ 488 eBioscience™ was obtained from Invitrogen (Carlsbad, CA, USA). FxCycle^TM^ PI/RNase staining solution and Dynamo SYBR Green qPCR Thermo DNAz I Thermo were obtained from ThermoFisher (Waltham, MA, USA). The RNAeasy Plus kit was obtained from Qiagen (Germantown, MD, USA) and the cDNA synthesis kit for RT-PCR from Applied Biosystems (Waltham, MA, USA).

### 2.2. Cell Lines

The LoVo cell line (colon adenocarcinoma cell line, Dukes’ type C, grade IV, ATCC^®^ CCL-229™) was obtained from ATCC (American Type Culture Collection). The doxorubicin-resistant subline LoVo/DX cell line was obtained by continuous in vitro exposure of LoVo cells to low doses of doxorubicin for 3 months [20]. The cells were cultured in DMEM/F12 medium, supplemented with 10% FBS, 2 mM L-glutamine, and 25 µg/mL gentamicin, at 37 °C in CO_2_-incubator. The cells were sub-cultured twice a week using the TrypLE^TM^ Express solution. 

### 2.3. Drug Solution

Celastrol and resveratrol were dissolved in DMSO as 10 mM stock solutions and stored at −20 °C. The working solutions were freshly prepared before each experiment by 10× dilution of stock solutions in an HBSS. The final DMSO concentration in the cell culture did not exceed 0.02%. 

### 2.4. Hoechst 33342 and Rhodamine 123 Accumulation Assay

The intracellular accumulation of Hoechst 33342 and rhodamine 123 was assessed by flow cytometry, as described in the literature [21,22]. Briefly, cells (1 × 10^6^) were resuspended in 2 mL of prewarmed HBSS in plastic Falcon tubes and the drug solution was added to the samples. After a short (5 min) pre-incubation, Hoechst 33342 (5 μg/mL final) or rhodamine 123 (5 µM final) solution was added, then samples were further incubated at 37 °C for 1.5 h (Hoechst 33342 assay) or 1 h (rhodamine 123 assay) with shaking every 30 min. Next, cells were washed with ice-cold HBSS (4 °C), and the cells’ pellet was resuspended in 1 mL of ice-cold HBSS, and immediately analyzed with CyFlow^®^ SPACE flow cytometer (Sysmex-Partec). Intracellular fluorescence measurements were performed using 365 nm laser excitation with 455/50 (BP) filter for Hoechst 33342 and 488 nm laser excitation with 536/40 (BP) filter for rhodamine 123. 

### 2.5. Side Population Assay

The SP analysis was performed according to the SP protocol described previously [20]. Cells were suspended in pre-warmed HBSS/2% FBS to the final cell density of 1 × 10^6^ cells/mL. The cells’ suspension was placed into plastic Falcon tubes and drug solution was added to the samples. After 5–10 min of incubation, Hoechst 33342 solution was added to the final concentration of 5 μg/mL. Samples were then incubated at 37 °C for 1.5 h with shaking every 30 min. Then cells were washed, resuspended with ice-cold HBSS, and analyzed on CyFlow^®^SPACE cytometer. The samples were kept on ice until flow cytometric analysis acquisition. The Hoechst dye was excited using UV laser (λ = 365 nm) and fluorescence was measured with a 455/50 BP filter (Hoechst blue) and a 630 LP filter (Hoechst red).

### 2.6. Detection of Intracellular ROS 

The DCF-DA assay was carried out with flow cytometry according to the protocol previously described [23]. The DCF-DA solution was prepared by dissolving in 100% ethanol and further 10× dilution in HBSS. Cells (1 × 10^6^/mL) were then resuspended in 1 mL of freshly prepared DCF-DA/HBSS solution (20 μM final) and the drug was immediately added to the samples for 1-h incubation at 37 °C in a CO_2_ incubator. Following incubation time, the cells were washed in cold HBSS and then immediately analyzed for intracellular content of DCF on CyFlow^®^SPACE flow cytometer (Sysmex-Partec) using 488 nm laser excitation with 536/40 (BP) filter. 

### 2.7. Immunofluorescence Staining for CD44 Detection

For immunofluorescence staining, cells were detached using Accutase™ Cell Detachment. Cells (1 × 10^6^) were resuspended in 80 µL of staining buffer (HBSS/2%FBS/2 mM EDTA), then 20 µL of FcR blocking reagent and 10 µL of FITC mouse anti-human CD44 were added. Samples were incubated for 20 min at 2–8 °C. Cells were washed with staining buffer and analyzed on the CyFlow^®^SPACE flow cytometer (Sysmex-Partec using) using 488 nm laser excitation with 536/40 (BP) filter.

### 2.8. Apoptosis and Necrosis Detection 

Cells were seeded in a 6-well plate and incubated with the drug for 24 h (37 °C, 5% CO_2_). For apoptosis and necrosis detection, cells were detached using Accutase™ Cell Detachment and stained using the FITC Annexin V Apoptosis Detection Kit, according to the manufacturer’s recommendation. Briefly, cells were resuspended in 100 μL of ice-cold 1x binding buffer and stained with 5 μL of Annexin V-FITC and 5 μL of PI for 15 min in the dark at room temperature. Samples were immediately analyzed with CyFlow^®^SPACE flow cytometer (Sysmex-Partec using) using 488 nm laser excitation with 536/40 (BP) filter for Annexin V-FITC and 675/20 (BP) for PI.

### 2.9. Cell Cycle Analysis

Cell cycle analysis was completed by quantification of DNA content using flow cytometry. For this purpose, cells were seeded in a 6-well plate and incubated with the drug for 24 h (37 °C, 5% CO_2_). Following incubation, the cells were removed from the wells using TrypLE™ Express solution and washed with cold HBSS. The cells were then fixed with ice-cold 70% ethanol and kept at −20 °C until analysis. For cells staining, FxCycle^TM^PI/RNase staining solution was used according to the manufacturer’s protocol. Briefly, 0.5 mL of staining solution was added to each sample and the samples were incubated for 15–30 min at room temperature, protected from light. Samples were acquired with CyFlow^®^SPACE flow cytometer (Sysmex-Partec using) using 488 nm laser excitation with 536/40 (BP) filter.

### 2.10. γH2AX Detection

DNA double-strand breaks were detected and quantified using phospho-histone H2A.X (Ser139) mouse monoclonal antibody (CR55T33) Alexa Fluor^®^ 488 eBioscience™, based on the protocol previously described [23]. For this purpose, cells were seeded in a 6-well plate and incubated with the drug for 24 h (37 °C, 5% CO_2_). Then, the cells were removed with TrypLE™ Express solution and washed twice with cold HBSS. The cells were fixed using 2% paraformaldehyde (PFA) for 10 min on ice. After two washing steps with cold HBSS/1% BSA, the cells were permeabilized with ice-cold 70% ethanol. The samples were kept in this solution for 5–7 days at −20 °C. Before staining with antibody, the cells were washed twice using HBSS/1% BSA. The cell pellets were resuspended in 100 μL of HBSS/1% BSA containing 2 μL of Phospho-histone H2A.X (Ser139) mouse monoclonal antibody (CR55T33) Alexa Fluor^®^ 488 eBioscience™ and incubated for 30 min in the dark. Then, the cells were washed with HBSS/1% BSA and analyzed by CyFlow^®^SPACE flow cytometer (Sysmex-Partec using) using 488 nm laser excitation with 536/40 (BP) filter.

### 2.11. Gene Expression Analysis

The level of mRNA for the analyzed genes was determined by real-time PCR. Cells were seeded in a 6-well plate and incubated with the drug for 24 h (37 °C, 5% CO_2_). Then, the cells were removed with TrypLE™ Express solution and washed twice with HBSS. RNA was extracted from cells using Qiagen RNAeasy Plus kit according to manufacturer’s recommendation and the residual DNA was removed with DNAse I. Quantitative and qualitative analysis of RNA was completed using Nanodrop (Thermoscientific, Waltham, MA, USA). The cDNA synthesis was completed using cDNA synthesis kit for RT-PCR. Gene expression was quantified by real-time PCR using Dynamo SYBR Green qPCR, according to the manufacturer’s recommendation. Step One Plus^TM^ Realy-Time PCR System (Applied Biosystems, USA) was used for carrying out the RT-PCR. GAPDH was used as a reference gene for normalization purpose. Changes in the levels of analyzed transcripts are presented as relative quantification (RQ) of the reference gene. Expression analysis of *BRCA*1, *PARP*1, and *SIRT*-1, -2, -3, 6 was carried out using the primer sequence listed in Table 1.

## 3. Results

### 3.1. Lovo/DX Colorectal Cancer Cell Lines Present Cancer Stem-like Properties

**Figure 2:**
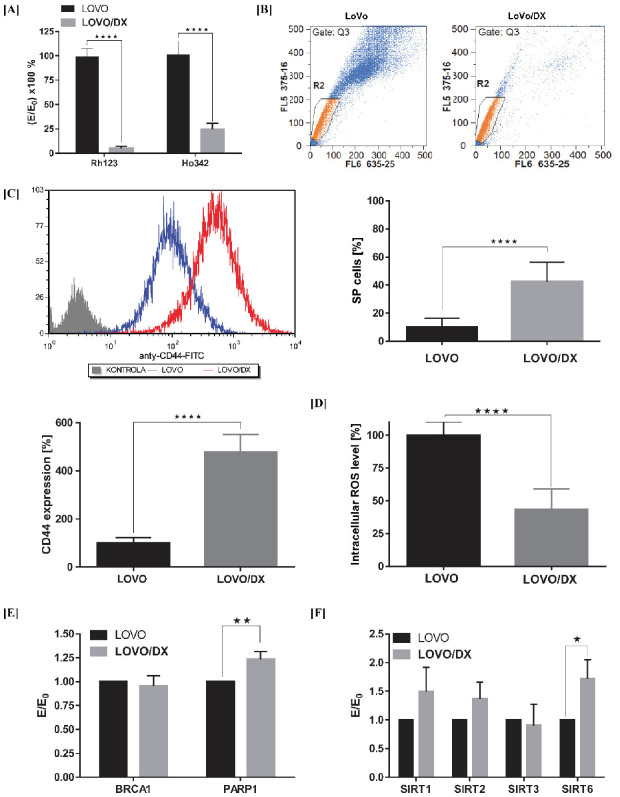
LoVo/DX cells display cancer stem cell–like properties compared to LoVo cells. (**A**) Intracellular accumulation of Rhodamine 123 (Rh123) and Hoechst 33342 (Ho342) fluorescent dyes. The results are expressed as E/E_0_ × 100%, where the MFI (mean fluorescent intensity) of Rh123 or Ho342 in LoVo/DX cells (**E**) was compared to the MFI in LoVo cells (E_0_); (**B**) Frequency of Side Population cells in LoVo and LoVo/DX cell-lines. Representative cytograms of flow cytometric analysis of Side Population cells. Side Population is defined as a subpopulation of cells that shows the lowest Ho342 content (a low-Ho342 fluorescence “tail” on dual wavelength of fluorescence emission: Hoechst red [630 nm] and Hoechst blue [455 nm]); (**C**) CD44 expression after immunofluorescence staining using FITC mouse anti-human CD44; (**D**) Intracellular ROS level measured by the means of DCF-DA assay; (**E**,**F**) mRNA expression level *of BRCA*1, *PARP*1, *SIRT*1, *SIRT*2, *SIRT*3, and *SIRT*6 genes obtained from real-time RT-PCR analysis. The results are expressed as E/E_0_, where the mRNA expression levels of the tested genes in LoVo/DX (E) cells were compared with the mRNA expression levels of these genes in LoVo (E_0_) cells. All data are the mean ± SD from at least three independent experiments. The significance of the differences was determined by Student’s *t*-test. * *p* < 0.05, ** *p* < 0.01, **** *p* < 0.0001.

Doxorubicin-resistant LoVo/Dx cells (a subline of LoVo cell line) were chosen as the in vitro cellular model of aggressive and resistant colon cancer enriched in CSCs. The LoVo cell line was derived from a metastatic tumor nodule of a patient diagnosed with adenocarcinoma of the colon. The LoVo/Dx cells are characterized by enhanced expression of MDR-associated transporters, MDR1 (P-gp), MRP1, BCRP, MDR3, and LRP, as compared with parental LOVO cells [24]. Increased functional activity of MDR proteins is known to be associated with maintaining the aggressive characteristics of cancer cells, i.e., CSCs. Here we show that LoVo/Dx cells display significantly higher activities of MDR transporters as detected by the efflux of fluorescent probes used as model MDR-substrates: Rhodamine 123 (Rh123) and Hoechst 33342 (Ho342). Due to efficient transport outside the LoVo/Dx cells, the intracellular accumulation of these probes was reduced by up to 5% for Rh123 and 25% for Ho342 (Figure 2A). In addition, the percentage of Side Population (SP) cells was significantly higher in the LoVo/DX cells compared to the LoVo cells (Figure 2B).

LoVo/DX cells highly overexpress the CD44 cell surface marker associated with colon cancer stemness (Figure 2C). CD44 is a hyaluronic acid receptor responsible for metastases by regulating cell-cell interaction, cell adhesion, and migration. CD44 plays a major role in maintaining a CSCs state, and targeted CD44 inhibits clonal formation and tumorigenicity in the xenograft model [25,26]. Moreover, CD44-variant (CD44v) isoform expression is associated with the upregulation of the ROS scavenging system, i.e., increased GHS synthesis is maintained by CD44v, which contributes to a lower level of basal ROS in the CSCs. As we demonstrated previously, the endogenous level of ROS in LoVo/DX cells is over 50% lower compared to parental LoVo cells [23] (Figure 2D).

CSCs have developed a constitutive upregulation of the DNA damage response (DDR). In colorectal cancer, higher PARP1 expression/activity levels have been associated with CSCs and with advanced/metastatic stages of colorectal cancer disease [27,28]. PARP1 and PARP3 have been demonstrated to contribute to key features characterizing CSCs including self-renewal capacity, metastasis, or resistance to DNA damaging therapies [29]. In addition, some evidence suggests that sirtuins, especially SIRT1 and SIRT2, might play essential roles in the maintenance and differentiation of various CSCs. Sirtuins regulate multiple cellular processes including DNA repair, aging, metabolism, cell cycle, and survival [19]. Here we show that the LoVo/DX cells exhibit an increased expression of PARP1, SIRT1, SIRT2, and SIRT6 genes compared to LoVo cells (Figure 2E,F).

All these findings indicates that LoVo/DX cells display important cancer stem cell–like characteristics and thus can serve as an experimental in vitro model of aggressive and resistant colon cancer.

### 3.2. Effect of RSV and CEL on Apoptosis and Necrosis Induction

The hallmarks of cancer cells and CSCs is the evasion of apoptosis. The ability to restore the susceptibility of cells to apoptosis is an important mechanism of anti-tumor activity. We first examined the pro-apoptotic properties of celastrol and resveratrol in both drug-sensitive and CSCs-enriched colon cancer cells. Pro-apoptotic or necrotic effects were assessed by double staining with Annexin V-FITC and PI dyes, which allows the identification of cells in the stage of early apoptosis (Annexin V-FITC+ and PI−), late apoptosis (Annexin V-FITC+ and PI+), and necrosis (Annexin V-FITC− and PI+).

**Figure 3:**
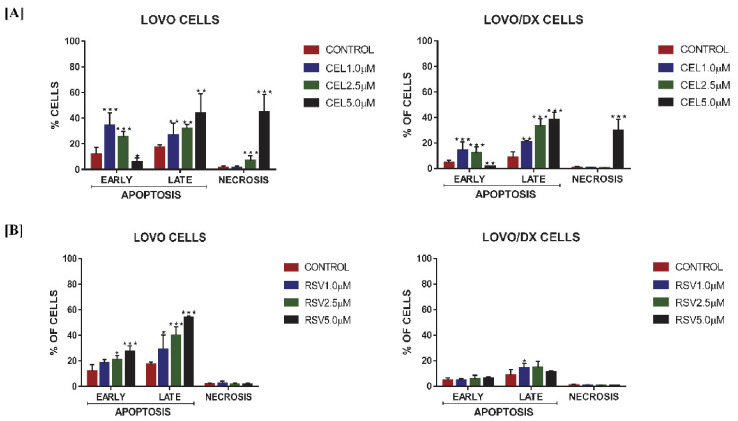
Effect of celastrol (CEL) (**A**) and resveratrol (RSV) (**B**) on the frequency of apoptotic and necrotic cells in LoVo and LoVo/DX cell cultures after 24 h of incubation. The cells were double-stained with Annexin V Alexa Fluo^®^r 488 and PI fluorescent dyes, and analyzed by flow cytometry. Control: cells incubated with the solvent (DMSO). The results are presented as a percentage of early apoptotic cells (Annexin V Alexa Fluor^®^ 488+ and PI−) and late apoptotic cells (Annexin V Alexa Fluor^®^ 488+ and PI+) and necrotic (Annexin V Alexa Fluor^®^ 488+ and PI+). The results are the mean ± SD of at least three independent experiments. The significance of the differences was determined by Student’s *t*-test. * *p* < 0.05, ** *p* < 0.01, *** *p* < 0.001.

As demonstrated in Figure 3A celastrol induces apoptosis in both LoVo and LoVo/DX cells. The frequency of apoptotic cells (in the early + late stage of apoptosis) increased up to 60% in LoVo cells and up to 45% in LoVo/Dx cells. With the increase in celastrol concentration, a decrease in early apoptotic cells accompanied by an increase of late apoptotic and necrotic cells was observed, indicating that colon cancer cells can pass through the apoptosis process fairly quickly, after being induced by celastrol. 

Resveratrol has a strong pro-apoptotic effect only on LoVo cells (Figure 3B). The number of apoptotic cells (both bearing characteristics of early and late apoptosis) raised to 80% in the dose-dependent manner. However, it showed only very little influence on LoVo/DX cells. 

### 3.3. Effect of RSV and CEL on Cell Cycle

**Figure 4:**
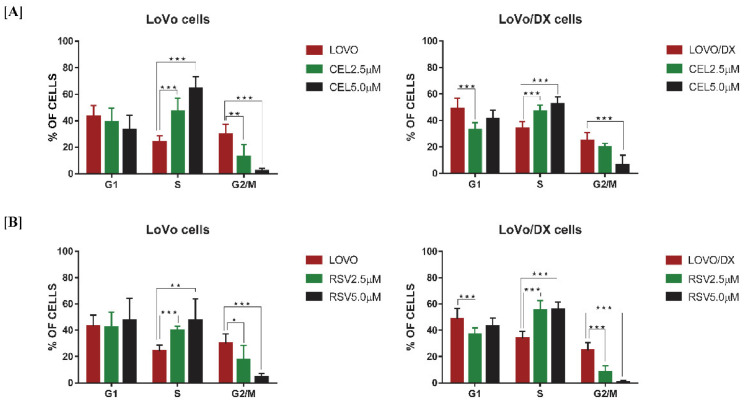
Effect of celastrol (CEL) (**A**); and resveratrol (RSV) (**B**) on cell cycle distribution in LoVo and LoVo/DX cell cultures after 24 h of incubation. The bar graphs show the percentage of cells in G1, S, and G2/M phases. Control comprises of cells incubated with solvent (DMSO). The results are the mean ± SD of at least three independent experiments. The significance of the differences was determined by Student’s *t*-test. * *p* < 0.05, ** *p* < 0.01, *** *p* < 0.001.

Further, we investigated whether induction of apoptosis by celastrol or resveratrol is associated with disruption of the cell cycle progression. Cell cycle arrest is often caused by the DNA damaging compounds, to induce cell senescence or apoptosis. Cell cycle distribution in LoVo and LoVo/DX cells after 24 h of treatment with the compounds is shown in Figure 4A (for celastrol) and 4B (for resveratrol). We found that increasing concentration of both polyphenols caused a significant increase in the cell population at the S phase along with a decrease in cell population in the G2/M phase. Celastrol blocks cell cycle progression more strongly in sensitive LoVo cells compared to LoVo/Dx cells, which correlates with its effect on apoptosis induction. Resveratrol-induced cell cycle arrest is comparable in both cell lines, however, it caused a slight pro-apoptotic effect in LoVo/Dx cells.

### 3.4. Effect of RSV and CEL on DNA Damage

**Figure 5:**
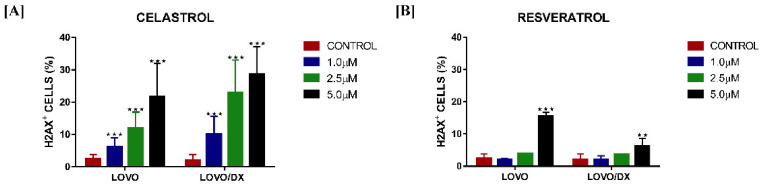
Impact of celastrol (CEL) (**A**) and resveratrol (RSV) (**B**) on the frequency of DNA double-strand breaks (γ-H2AX+ cells) in LoVo and LoVo/DX cell cultures after 24 h of incubation. γ-H2AX+ cells were detected using phospho-histone H2A.X (Ser139) monoclonal antibody (CR55T33) Alexa Fluor^®^ 488. Control constitutes cells incubated with solvent (DMSO). The results are the mean ± SD of at least three independent experiments. The significance of the differences was determined by Student’s *t*-test. ** *p* < 0.01, *** *p* < 0.001.

To determine if cell apoptosis and cell cycle arrest are connected to the induction of DNA damage, we measured the frequency of cells showing unrepairable DNA DSBs after incubation with the compounds. Celastrol has shown to be a potent DNA-damaging compound, leading to a significant concentration-dependent increase in DSBs (Figure 5A). Interestingly, colon cancer cells enriched with CSCs (LoVo/Dx) were more sensitive to the DNA-damaging effects of celastrol. Resveratrol, at 5 µM, effectively induced DSBs in LoVo cells, with a smaller effect in LoVo/Dx cells (Figure 5B). 

### 3.5. Effect of RSV and CEL on BRCA and PARP Gene Expression and ROS Generation 

**Figure 6:**
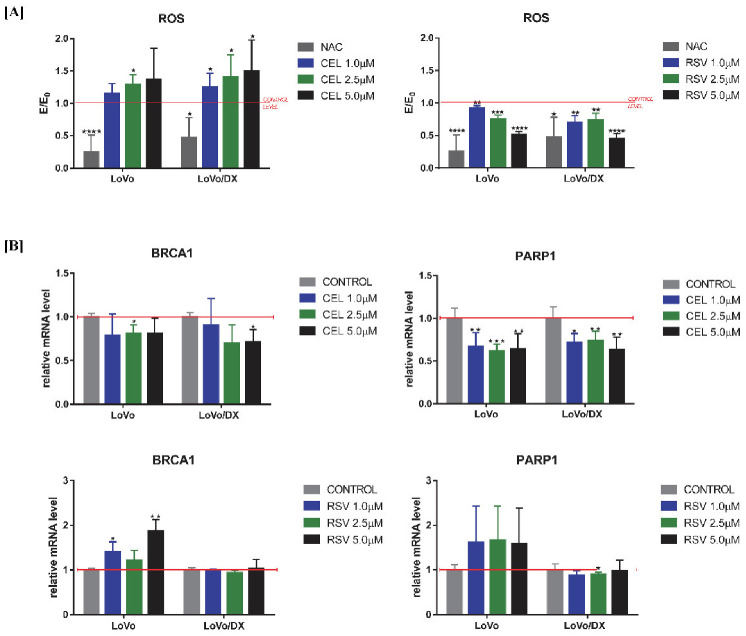
Impact of celastrol (CEL) and resveratrol (RSV) on intracellular ROS level (**A**); and mRNA expression of BRCA1 and PARP1genes (**B**) in LoVo and LoVo/DX cell cultures after 24 h of incubation. Intracellular ROS level measured by the means of DCF-DA assay. The MFI (mean fluorescent intensity) of DCF obtained in the presence of celastrol or resveratrol (E) was compared to the relevant control (E_0_), i.e., cells incubated in the presence of the solvent (DMSO). The mRNA expression level of BRCA1 and PARP1 genes was obtained from real-time RT-PCR analysis. The results are the mean ± SD of at least three independent experiments. The significance of the differences was determined by Student’s *t*-test. * *p* < 0.05, ** *p* < 0.01, *** *p* < 0.001, **** *p* < 0.0001.

Irreversible DNA damage is often the cause of oxidative stress and an ineffective DNA damage response. The *PARP*1 and *BRCA*1 genes are involved in the response to cellular damage through activation of specific DNA repair processes. We, therefore, investigated the effects of celastrol and resveratrol on ROS generation (Figure 6A) and *PARP*1/*BRCA*1 genes expression (Figure 6B) in both LoVo and LoVo/Dx cells. We found that celastrol significantly increased intracellular levels of ROS and impaired the ability of cells to cause ROS-induced DNA damage, by reducing the expression of both *PARP*1 and *BRCA*1 genes. In contrast, resveratrol showed to have strong, concentration-dependent, antioxidant effects in both cell lines. Surprisingly, resveratrol caused overexpression of *BRCA*1 and *PARP*1 genes in drug-sensitive cells and showed a lack of (*BRCA*1), or marginally decreasing (*PARP*1), effect on LoVo/Dx cells.

### 3.6. Effect of RSV and CEL on SIRT Gene Expression 

**Figure 7:**
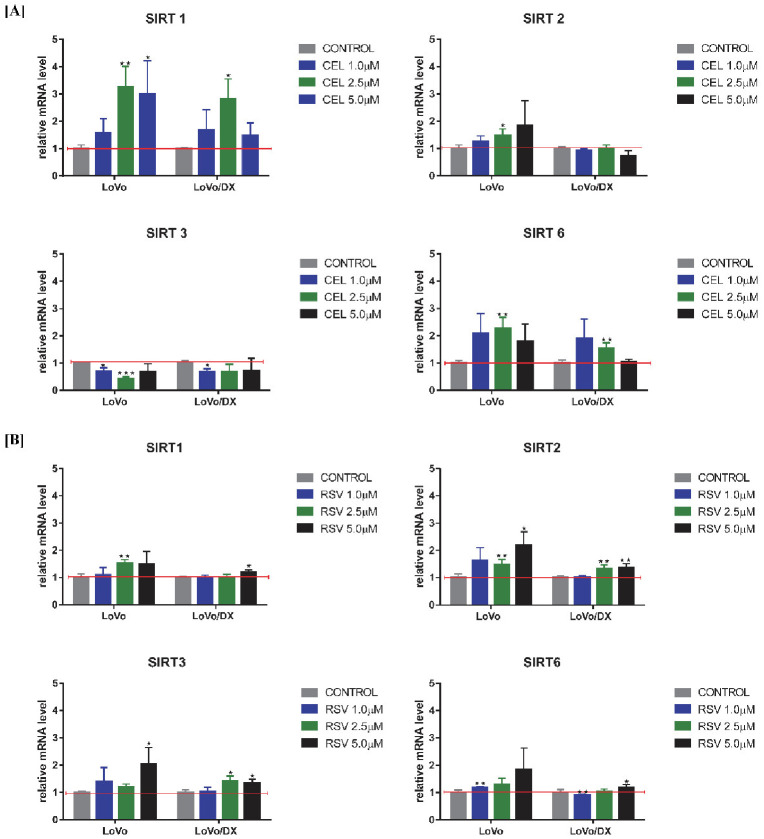
Impact of celastrol (CEL) (**A**) and resveratrol (RSV) (**B**) on mRNA expression of *sirtuins* (*SIRT*) genes in LoVo and LoVo/DX cell cultures after 24 h of incubation. The mRNA expression level was obtained from real-time RT-PCR analysis. The results are the mean ± SD of at least three independent experiments. The significance of the differences was determined by Student’s *t*-test. * *p* < 0.05, ** *p* < 0.01, *** *p* < 0.001.

Further, we evaluated the influence of celastrol and resveratrol on the expression of *SIRT* 1, 2, 3, and 6 genes. Sirtuins play various roles in cancer by affecting the response to genomic instability, regulating cancer-associated metabolism, and modifying the tumor microenvironment, and they are implicated in cell cycle, cell division, and transcription regulation. As shown in Figure 7 and Table 2, celastrol increased the mRNA expression of *SIRT* 1, 2, 6 in LoVo cells, and *SIRT* 1, 6 in LoVo/Dx cells, whereas it reduced the mRNA expression of *SIRT* 3 in both cell lines. Resveratrol increased the mRNA expression of all tested *sirtuins* in LoVo cells and *SIRT* 2 and 3 in LoVo/Dx cells. The mRNA expression of *sirtuins* 1 and 6 was only slightly increased at a resveratrol concentration of 5 µM. 

## 4. Discussion

The recovery rate in patients with metastatic colorectal cancer (CRC) remains low and declines with successive lines of treatment due to the development of resistance to current therapies. Colorectal cancer stem cells (CSCs), present in the tumor mass, are responsible for this phenomenon, making them attractive potential targets for the treatment of CRC. Celastrol and resveratrol are very promising phytochemicals for cancer therapy due to their low or no toxicity and pleiotropic activity that enables them to interact with various biological targets. In the present study, the potential anticancer mechanisms of both compounds against metastatic colon cancer cells and the capacity to eradicate CSCs were investigated. For this purpose, two colon cancer cell lines were used: LoVo cells which were derived from metastatic nodules of colon adenocarcinoma (with standard sensitivity to drugs), and LoVo/Dx cells (doxorubicin-resistant subline of LoVo cell line). LoVo/DX cells were chosen as the in vitro cellular model of aggressive and resistant colon cancer, enriched in CSCs. As was previously demonstrated, LoVo/Dx cells highly overexpress some MDR proteins [24]. In addition, here we showed that LoVo/Dx cells exhibit several other CSC-like characteristics, such as amplified drug efflux capacity; high expression of CD44, a CSCs-biomarker; increased capacity to DNA repair through the PARP mechanism; and decreased levels of ROS. CRCs are resistant to DNA damaging therapies by regulation of the cell cycle, increasing DNA repair capacity, and effective scavenging of ROS [30]. Moreover, the percentage of CSCs, estimated from the size of SP cells, was significantly higher compared to parental LoVo cells. Further, we found that LoVo/Dx cells expressed SIRT 1, SIRT 2, and SIRT6 mRNA, but not SIRT 3, more strongly than LoVo cells. A growing body of evidence indicates the involvement of SIRTs in epigenetic regulation of important tumor processes, such as proliferation, cellular metabolic reprogramming, invasion, and metastasis [18]. It was also proposed that sirtuins, especially SIRT1 and SIRT2, might play essential roles in the maintenance and differentiation of various cancer stem cells [19]. 

Malignant neoplasms consist of a subpopulation of cells that exhibit varying degrees of chemoresistance. Chemotherapy mostly targets highly proliferating cells, while CSCs are a rare quiescent cell population, in the resting stage of the cell cycle, easily escaping the toxic effects of drugs. Apoptosis resistance is a common feature of cancer and CSCs cells [30], and the induction of apoptotic death is particularly important for successful cancer therapy. Here we found that celastrol was able to significantly reduce the death resistance of CSC-like cells (LoVo/Dx cells) and colon cancer cells (LoVo) through apoptosis and necrosis induction. It seems that LoVo cells are slightly more susceptible to the effects of celastrol, which correlates with their drug-sensitive phenotype. One of the mechanisms involved in celastrol-induced apoptotic/necrotic death is related to boosting ROS production. We previously demonstrated that celastrol induces a significant increase in cytoplasmic and mitochondrial ROS levels after 1-h cultivation with LoVo/Dx cells [23]. Moreover, as we have shown in this study, a higher amount of ROS was also detected after 24 h of incubation with lower celastrol concentrations in both LoVo and LoVo/Dx cells. Although the basic level of free radicals in CSC-like LoVo/Dx cells is lower than in LoVo, these cells respond more strongly to the pro-oxidative effects of celastrol. This may suggest that celastrol has the potential to disturb the redox status of CSCs that control ROS production and scavenging to maintain a protective low level.

The pro-apoptotic effect of resveratrol depends on the cancer cell phenotype. Takashina M. et al. suggested that resveratrol induces apoptosis more effectively in leukemic cells than in solid tumor cells. The authors showed that resveratrol had a slight effect on cell viability in Caco-2, HCT116, and SW480 colon cancer cells [31]. However, other authors reported that resveratrol and its analogues can inhibit cell viability both in primary (Colo-320) and metastatic (Colo-741, LoVo, DLD-1) colon cancer cells [32,33]. In our studies, resveratrol strongly induced apoptosis of LoVo cells and had an only marginal effect on LoVo/DX cells. It is unclear why CSC-like cells escape the pro-apoptotic effects of resveratrol. Prolonged treatment with high concentrations may be necessary to cause apoptotic cell death, as in some cases resveratrol appears to be a slow inducer of apoptosis [34]. Moreover, resveratrol did not induce necrotic cell death in both tumor cell lines, possibly due to its protective antioxidant properties.

Blocking cell cycle progression halts the proliferation of cancer cells and is an important mechanism of cytotoxicity induced by a cytostatic drug. Previously, we have shown that celastrol induces cell cycle arrest in the S phase in LoVo/Dx cells [23]. Here we demonstrated that celastrol blocks cell cycle progression more strongly in sensitive LoVo cells compared to LoVo/Dx cells, which correlates well with its effects on apoptosis induction. Resveratrol also arrests the cell cycle by the accumulation of the cells in the S phase, in both cell lines. However, it has a minor proapoptotic effect in LoVo/Dx cells, which indicates various mechanisms of anti-tumor activity in sensitive and CSC-like colon cancer cells. In agreement with our findings, Delmas D. et al. also showed that resveratrol-induced cell growth arrest is associated with S-phase cell accumulation in the SW480 colon cancer cell line. In addition, resveratrol completely blocked SW480 cell cycle progression during the S^G2/M transition. The cell cycle inhibitory properties of resveratrol in SW480 cells could be attributed to the accumulation of inactive forms of key cell cycle regulators rather than to the inhibition of DNA synthesis [35]. 

**Figure 8:**
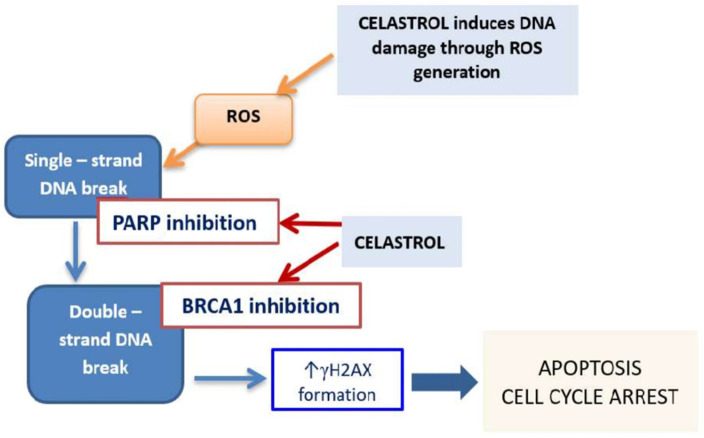
Possible mechanism of anticancer activity of celastrol on colon cancer cells.

In the normal course of the cell cycle, the S phase allows for proper DNA replication. Halting of the cell cycle progression at the S phase may be the response to DNA damage, thereby allowing time for DNA repair. In the case of cancer cells, drug-induced DNA double-strand breaks (DNA DSBs) frequently result in cell cycle arrest at the S phase and induction of apoptosis [36]. In our study, we demonstrated that celastrol is a potent DNA damaging compound, leading to a significant increase in unrepairable DNA DSBs. Interestingly, CSC-like LoVo/Dx cells were more sensitive to the DNA damaging effects of celastrol, probably due to the higher celastrol-induced intracellular ROS level, compared to LoVo cells. In addition, we found that celastrol impaired the ability of colon cancer cells to repair DNA by reducing the expression of both *PARP*1 and *BRCA*1 genes (Figure 8).

**Figure 9:**
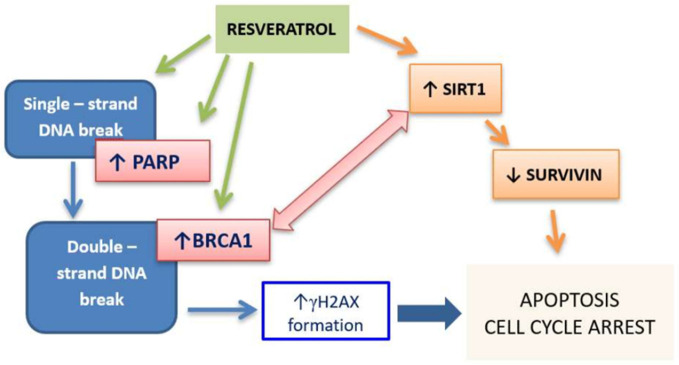
Possible mechanism of anticancer activity of resveratrol on LoVo colon cancer cells.

Several reports indicated the ability of resveratrol to induce DNA DSBs [37,38]. Demoulin B. et al. demonstrated that treatment of HCT 116 colon cancer cells with resveratrol caused the formation of γ-H2AX foci proportionally to the concentration of the polyphenol, and this effect was ROS-independent [37]. In our study, resveratrol induced DSB in both cell lines, however, γ-H2AX expressing cells started to appear at 2.5 µM and increased significantly at 5 µM. The effect was stronger in LoVo cells compared to LoVo/Dx cells which correlates with high pro-apoptotic activity in these cells. It should be pointed out that resveratrol in both cell lines, LoVo and LoVo/Dx, decreased intracellular ROS level, thus the mechanism by which resveratrol caused DNA damage is not dependent on ROS. Surprisingly, in drug-sensitive LoVo cells resveratrol caused overexpression of *BRCA*1 and *PARP1* genes which does not match with its potent cytotoxic activity. It was reported that the increased expression of *BRCA*1 was associated with *SIRT*1 activation that alters H3 acetylation [39]. In line with other studies, we showed that resveratrol increases the expression of the *SIRT*1 gene in LoVo cells [40,41]. SIRT 1 negatively regulates survivin expression [40]. Survivin is a member of the inhibitor of apoptosis (IAP) family and its overexpression in cancer cells contributes to their resistance to apoptotic stimuli and chemotherapeutic therapies. It might be therefore suggested that resveratrol, by epigenetic mechanism i.e., increasing *SIRT*1 gene expression, contributes to overcoming apoptosis resistance in LoVo colon cancer cells (Figure 9). In CSC-like LoVo/Dx cells, resveratrol did not affect the *BRCA*1, *PARP*1, and *SIRT*1 genes, which, with little pro-apoptotic effect, suggests a different mechanism of its cytotoxic action in those cells.

SIRT1 deacetylates many of the non-histone proteins that are involved in cell growth, apoptosis, cell senescence, and tumorigenesis. Several recent studies have provided evidence that SIRT1 serves as a tumor suppressor and that overexpression of SIRT1 attenuates cancer formation both extends lifespan and inhibits cancer formation. In colon cancer, upregulation of SIRT1 reduced cancer cells proliferation through the ability of SIRT1 to deacetylate β-catenin and promote cytoplasmic localization of the nuclear-localized oncogenic form of β-catenin. In addition, SIRT1 deacetylates the RelA/p65 subunit of NF-κB and sensitizes cells to TNFα-induced apoptosis by inhibiting the transactivation potential of the RelA/p65 protein [42]. In our research, celastrol increased the expression of the *SIRT*1 gene in LoVo and LoVo-Dx cells, which may contribute to its antitumor activity by disrupting the β-catenin and NF-κB pathways. As it was previously shown, β-catenin mediates the apoptosis induction effects of celastrol in HT29 cells [43] and celastrol is a well-known NF-κB inhibitor. Resveratrol in LoVo cells also increased *SIRT*1 expression, however, it has very slight effect on CSCs like LoVo/DX cells. Buhrmann C. et al. reported that in CRC cell lines (HCT116 and SW480) resveratrol can suppress tumorigenesis, at least in part, by targeting SIRT1 and suppressing NF-κB activation [44].

Although there are potentially two opposing roles of SIRT2 as a tumor suppressor and a promotor [45], there is increasing evidence that overexpression of SIRT2 significantly inhibits CRC proliferation, migration, and invasion [46,47]. For example, Ozden O. and Parkin S-H demonstrated that SIRT2 affects the expression of oncogenic FOXM1 in HCT116 colon cancer cells leading to a decrease in the number of colony formations and proliferation. Thus SIRT2-activating molecules may be used to downregulate FOXM1 to diminish the proliferative potential of cancer cells [48]. Moreover, overexpression of SIRT2 induces cell cycle S phase arrest of normal cells and colon cancer cells [47]. In our study, celastrol and RSV are able to increase *SIRT*2 expression in sensitive LoVo cells, which could possibly be related to the S phase arrest of cells. However, in CSC-like LoVo/DX cells, celastrol did not change the *SIRT*2 levels, and resveratrol increased very slightly the *SIRT*2 expression. It should be pointed out that LoVo/DX cells present higher basal *SIRT*2 levels compared to LoVo cells. Thus, it remains possible that SIRT2’s functions depend on different tissue contexts, an abundance of different SIRT2 downstream targets, and factors that regulate SIRT2. 

**Figure 10:**
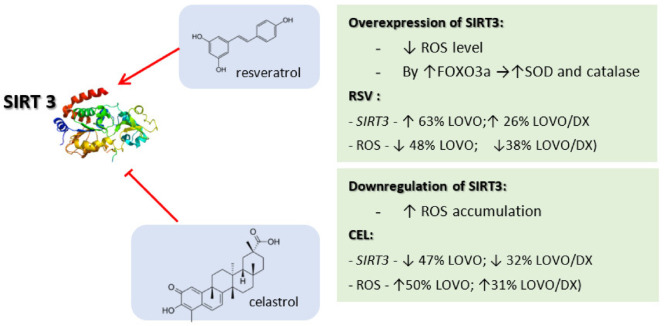
The interplay between SIRT3, ROS, and celastrol/resveratrol effects on colon cancer cells.

Accumulating evidence has recently demonstrated that SIRT3 has a key role in cancer, influencing cell death by targeting a series of key modulators and their relevant pathways in cancer. However, depending on the cell type and tumor type, SIRT3 may function as either oncogene or tumor suppressor. Moreover, SIRT3 is an important mitochondrial deacetylase, responsible for the maintenance of appropriate mitochondrial function by limiting oxidative stress and reducing reactive oxygen species (ROS) production with a decrease in mitochondrial membrane potential [49]. Here, we showed that celastrol decreased *SIRT*3 gene expression in both drug-sensitive LoVo cells and CSC-like LoVo/Dx cells, which corresponds to its ROS-generating activity. It can therefore be suggested that the pro-oxidative activity of celastrol is mediated by an epigenetic mechanism involving the reduction of SIRT3-dependent regulation of ROS production. In contrast, resveratrol increased the expression of the *SIRT*3 gene, which may be related to decreased intracellular ROS levels. Thus, resveratrol-induced *SIRT*3 upregulation might be responsible for its antioxidant effect (Figure 10).

SIRT6 was proposed to serve as a prognostic indicator and potential therapeutic target in colon cancer [50]. SIRT6 is pathologically downregulated in colon cancer and its low expression is associated with a poor prognosis and more aggressive progression of this disease [50,51]. In contrast, over-expression of SIRT6 inhibits cell proliferation, invasion, and migration, and enhances cell apoptosis by upregulating PTEN and downregulating AKT1 expression [50]. Targeting SIRT6 using a small-molecule activator is an attractive therapeutic strategy for CRC that is investigated in various CRC models [51]. Here we show that celastrol increases *SIRT*6 expression in both sensitive and CSCs-like cells, which correlates well with its anti-cancer effects. Resveratrol is a better activator of *SIRT*6 in drug-sensitive LoVo cells, which corresponds to its greater anti-cancer properties in these cells.

## 5. Conclusions

In conclusion, we have shown that celastrol and resveratrol exert an anti-tumor activity against metastatic LoVo cells and cancer stem-like LoVo/DX cells through various mechanisms, including induction of harmful DSB, apoptosis, or cell cycle arrest. It appears that epigenetic modification governed by *sirtuin* genes may be related to some of the anti-tumor effects of both compounds. Celastrol has been shown to be a more potent anti-tumor compound against colon cancer, capable of attenuating CSC-like cells at the molecular and cellular levels. It can therefore be proposed as a potential agent for combating aggressive types of colon cancer. In contrast, resveratrol has a much greater effect on colon cancer cells expressing standard sensitivity to anticancer drugs than on CSC-like cells, suggesting its potential use in patients with less aggressive forms of the disease.

## Figures and Tables

**Table 1:** Sequences of the primers used to analyze gene expression.

Target Gene	Forward Primer	Reverse Primer
*GAPDH*	5′-AGGTCGGAGTCAACGGAT-3′	5′-TCCGGAAGATGGTGATG-3′
*BRCA1*	5′-GGAAATTCTGAGGCAGGTAT-3′	5′-CTGGGATTACAGGCGTG-3′
*PARP1*	5′-CAACAGAAGTAC GTGCAA5′-	5′-GGTCAA TCATGC CTAGC-3′
*SIRT1*	5′-CCAAGCAGCTAAGAGTAAT-3′	5′-TTTCCATCTGTTCAGCAA-3′
*SIRT2*	5′-CAGAGTCATCTGTTTGGT-3′	5′-GGTACTTCTCTAGGTTGTCATA-3′
*SIRT3*	5′-GGAGCTGCTCATCAACC-3′	5′-TTCTGTCCAGCCCAGAA-3′
*SIRT6*	5′-GAAGAATGTGCCAAGTGTAA-3′	5′-GGAGTCCTCCCAGTCTA-3′

**Table 2:** Maximal increase/decrease of mRNA expression of tested sirtuins’ genes after 24 h of incubation with celastrol or resveratrol.

CELASTROL	LoVo Cells	LoVo/DX Cells
*SIRT1*	↑ 226% (2.5 µM)	↑ 181% (2.5 µM)
*SIRT2*	↑ 84% (5 µM)	↓ 27% (5 µM)
*SIRT3*	↓ 57% (2.5 µM)	↓ 32% (1 µM)
*SIRT6*	↑ 126% (2.5 µM)	↑ 90% (1 µM)
RESVERATROL	LoVo Cells	LoVo/DX Cells
*SIRT1*	↑ 52% (2.5 µM)	↑ 18% (5 µM)
*SIRT2*	↑ 118% (5 µM)	↑ 37% (5 µM)
*SIRT3*	↑ 105% (5 µM)	↑ 40% (2.5 µM)
*SIRT6*	↑ 85% (5 µM)	↑ 19% (5 µM)

↑ increase; ↓ decrease.

## Data Availability

The data presented in this study are available in the article.
